# Prevalence and determinants of insufficient work ability in older HIV-positive and HIV-negative workers

**DOI:** 10.1007/s00420-015-1108-0

**Published:** 2016-01-08

**Authors:** Lisanne M. Möller, Ronald Brands, Judith K. Sluiter, Judith Schouten, Ferdinand W. Wit, Peter Reiss, Maria Prins, Ineke G. Stolte

**Affiliations:** Cluster Infectious Diseases, Department of Research, Public Health Service, Amsterdam, Nieuwe Achtergracht 100, 1018 WT Amsterdam, The Netherlands; Dutch HIV Association, Amsterdam, The Netherlands; STI AIDS, Amsterdam, The Netherlands; Coronel Institute of Occupational Health, Academic Medical Center, Amsterdam, The Netherlands; Department of Global Health, Academic Medical Center, Amsterdam Institute for Global Health and Development, Amsterdam, The Netherlands; Department of Neurology, Academic Medical Center, Amsterdam, The Netherlands; Department of Internal Medicine, Division of Infectious Diseases, Center for Infection and Immunity Amsterdam (CINIMA), Academic Medical Center, Amsterdam, The Netherlands; HIV Monitoring Foundation, Amsterdam, The Netherlands

**Keywords:** Work ability, HIV, Aging, Employment, Comorbidity

## Abstract

**Purpose:**

To explore whether the prevalence and determinants of insufficient work ability (WA) of older HIV-positive workers differ from a comparable group of HIV-negative workers.

**Methods:**

Cross-sectional data from 359 HIV-negative and 264 HIV-positive middle-aged individuals (45–65 years) participating in paid labor, collected within the AGE_h_IV Cohort Study between October 2010–September 2012, were selected. Data were collected by self-administered questionnaires and physical examination. Participants self-rated their current WA, ranging from 0 to 10. WA was dichotomized into insufficient (<6) and sufficient (≥6). Using univariable and multivariable logistic regression, we studied the independent effect of HIV status on insufficient WA and determinants of insufficient WA.

**Results:**

Overall, 8 % of participants reported insufficient WA (HIV-positive 9 vs. HIV-negative 7 %, *P* = 0.20). Twice as many HIV-positive as HIV-negative individuals were declared partly unfit for work (6 vs. 3 %, *P* = 0.02). HIV status itself was not associated with WA in univariable and multivariable analyses. Multivariable analyses revealed that low educational level, working fewer hours, being partly unfit for work, experiencing a high need for recovery after work, staying home from work ≥2 times in the past 6 months, and reporting depressive symptoms were associated with insufficient WA, independent of HIV status.

**Conclusions:**

HIV-positive individuals aged 45–65 years participating in paid labor seem to function as well at work as HIV-negative individuals. HIV-positive participants were more often formally declared partly unfit for work, but percentages were low in both groups. Knowledge of determinants of insufficient WA may help employers and professionals to optimize WA.

## Introduction

The availability of combination antiretroviral treatment (cART) from 1996 onward completely changed the HIV epidemic, transforming HIV from a fatal disease into a chronic disorder. This has led to a growing and aging group of people living with HIV (PLWH) (Hasse et al. 2011). Evidence suggests that PLWH experience more age-related comorbidity than the general population (Hasse et al. 2011; Goulet et al. 2007), and at an earlier age (Effros et al. 2008; Guaraldi et al. 2011; Pathai et al. 2014), which could partly explain why HIV-positive individuals on average still have a shorter life expectancy than HIV-negative individuals (Antiretroviral Therapy Cohort Collaboration 2008). These age-associated comorbidities may also influence the social participation of PLWH. In general, people with chronic disorders are less often employed than others (Baanders et al. 2002), and if they are employed, they report difficulties in meeting physical, psychosocial, and environmental work demands (Lerner et al. 2000). Several comorbidities, such as hypertension, diabetes, and depression, have been shown to increase the risk of work cessation among PLWH (Dray-Spira et al. 2012), and despite the success of cART, studies consistently show high unemployment rates among PLWH (Dray-Spira and Lert 2007; Ezzy et al. 1999; Rabkin et al. 2004). A recent study in the Netherlands found participation rates of 63 % for PLWH between 45 and 65 years and 79 % among HIV-negative people in the same age range (Stolte et al. 2013). Whether working PLWH experience problems in functioning at work is unknown. It is important to identify problems people experience at work at an early stage to prevent job loss. Work ability, a measure of the degree to which someone is physically and mentally able to cope with the demands at work (Tuomi et al. 1998; Ilmarinen 1991), has been shown to predict later disability and work loss (Tuomi et al. 1991; Salonen et al. 2003; Burdorf et al. 2005). Insight into the factors that are associated with insufficient work ability might help employers and occupational physicians prevent work disability and premature retirement. This study aimed (1) to examine whether the prevalence of insufficient work ability in older (≥45 years) HIV-positive workers differs from a comparable group of HIV-negative workers and (2) to explore which determinants are associated with insufficient work ability. We hypothesized that the prevalence of insufficient work ability would be higher among HIV-positive than among HIV-negative workers.

## Methods

### Study design

For this study, baseline data from the AGE_h_IV Cohort Study were used, details of which have been previously reported (Schouten et al. 2014). In short, this ongoing prospective cohort study on comorbidity and aging with HIV started in 2010 and has included 598 HIV-positive and 550 HIV-negative individuals aged 45 years or older, of which 539 HIV-positive and 525 HIV-positive participants completed the standardized study questionnaire at the first visit. HIV-positive participants were recruited at the HIV outpatient clinic of the Academic Medical Center in Amsterdam and HIV-negative controls through the ongoing Amsterdam Cohort Studies on HIV/AIDS and among visitors of the sexual health clinic of the Amsterdam Public Health Service.

After obtaining informed consent, participants underwent a standardized screening for comorbidities and completed an extensive questionnaire to collect data on socio-demographics, medical history, medication use, lifestyle, quality of life, depression, sexual orientation/behavior/dysfunction, cognitive complaints, and work-related factors. Detailed information concerning HIV infection and ART history was obtained from the database of the Dutch HIV Monitoring Foundation. The study protocol was approved by the local ethics review committee and was registered at www.clinicaltrials.gov (identifier: NCT01466582).

For the present study, participants who participated in paid labor (currently working as an employee, independent entrepreneur, or partially working/partially disabled) and were of working age (45–65 years) were included. In total, 295/539 HIV-positive and 376/525 HIV-negative individuals met these inclusion criteria. Of these participants, 31 HIV-positive and 17 HIV-negative individuals were additionally excluded because of incomplete data, resulting in a study population of 264 HIV-positive and 359 HIV-negative participants.

### Variables

The main outcome of perceived work ability was measured using the central item of the Work Ability Index (WAI) (Tuomi et al. 1998; Ilmarinen 1991), in which participants were asked to rate their current work ability related to their lifetime best. The work ability score ranged from 0: not able to work, to 10: best self-perceived work ability ever. This single-item measure has been shown to be a good representation of the WAI (El Fazzi et al. 2013; Sell et al. 2009). Work ability was dichotomized into insufficient (<6) and sufficient (≥6), in analogy with the school rating system in the Netherlands (Ruitenburg et al. 2012).

Educational level was divided into low (education until 16 years), middle (education until 18 years), and high (college degree or higher). Sexual preference was categorized as homosexual/bisexual men, heterosexual men, and women. Ethnic background was dichotomized into Dutch and non-Dutch and marital status into married or cohabiting versus never married, divorced, or widowed. Lifestyle factors included physical activity, smoking, alcohol use, and drug use. Physical activity was defined according to Dutch healthy physical activity guidelines (‘Combinorm’): moderate physical activity ≥5 days/week for ≥30 min or heavy physical activity at least twice a week for ≥20 min (Ooijendijk et al. 2007). Smoking was quantified in pack years, with one pack year equal to smoking 20 cigarettes (one pack) a day for 1 year. Alcohol use was dichotomized into heavy daily drinking (≥4 drinks a day for women and ≥6 drinks a day for men) versus non-heavy daily drinking, and drug use was dichotomized into daily to monthly use of cannabis/cocaine/ecstasy and less often than daily to monthly use. Work-related factors were self-reported work type (paid employment, entrepreneur, or partly unfit for work), weekly work hours, lack of work-related recovery opportunities, and need for recovery after work. Lack of work-related recovery opportunities measures situational characteristics, both on and off the job, that allow workers to recuperate from work (e.g., ‘Can you interrupt your work if you find it necessary to do so?’). It was measured on a validated nine-item scale (van Veldhoven and Sluiter 2009), with a four-point format (always, often, sometimes, and never) as answering categories. Total scores were calculated and divided into tertiles (low ≤ 7, medium 8–12, high ≥ 13), with a high score implicating little opportunities to recover from work. Need for recovery after work (e.g., ‘Generally, I need more than an hour before I feel completely recuperated after work’) was measured with the need for recovery scale (van Veldhoven and Broersen 2003). This scale quantifies the difficulties workers experience in recovering from work with 11 items with a yes/no format. Total scores were calculated and divided into tertiles (low ≤ 1, medium 2–5, high ≥ 6), with a high score implicating a high need for recovery after work.

Age-associated comorbidities were measured via both questionnaire and physical examination. Comorbidity was defined as having a chronic disease, based on self-reported past disease, current disease, and medication use, combined with the outcomes of the physical examination. Details of the physical examination and the definition of comorbidities are described elsewhere (Schouten et al. 2014). The number of comorbidities was categorized into 0, 1, 2, or ≥3 comorbidities. Depressive symptoms were measured with the nine-item PHQ-9 scale (Kroenke et al. 2001), a validated depression screening tool based on the Diagnostic and Statistical Manual of Mental Disorders, Fourth Edition (DSM IV) criteria for major depressive disorder. Total scores were calculated and dichotomized into no or mild symptoms (<10), and moderate to severe depressive symptoms (≥10) (Kroenke et al. 2001). Self-reported absenteeism was measured by the frequency of staying home from work due to sickness in the past 6 months, categorized into never, 1 time, and ≥2 times.

HIV-related factors were time since HIV diagnosis, time since starting ART, mean CD4 cell count in the 12 months prior to study enrollment (cells/mm^3^), nadir CD4 count, prior clinical AIDS, undetectable viral load in the 12 months prior to enrollment, and having experienced work-related stigma. Prior clinical AIDS was defined as having had a previous AIDS-defining condition following the US Centers for Disease Control and Prevention classification. Experienced work-related stigma was measured by asking participants whether they were ever refused a job or discriminated at work because of their HIV positivity.

### Statistical analyses

Differences between HIV-positive and HIV-negative participants were examined with Chi-square tests for categorical data and Wilcoxon rank-sum tests for continuous data. Univariable associations between potential determinants and work ability were assessed with logistic regression analyses. Collinearity between possible determinants was measured with a Spearman correlation coefficient, with multicollinearity defined as a correlation >0.7. Due to the high number of variables, multiple multivariable models were used to explore possible determinants of work ability. HIV status and age were a priori included in all models. In model 1, all socio-demographic variables with a *P* value <0.1 in univariable analyses were included. Model 2 included lifestyle factors with a univariable *P* value <0.1, corrected for the socio-demographic factors from model 1. In models 3 and 4, work-related factors and factors on absenteeism and comorbidities with a univariable *P* value <0.1 were, respectively, included, corrected for socio-demographics. In addition, separate univariable and multivariable analyses were performed among HIV-positive participants only, to study the effect of HIV-related variables. This multivariable model included all socio-demographic variables from model 1 and HIV-related variables with a univariable *P* < 0.1. All analyses were performed with Stata Software version 11.2 (Stata Intercooled, College Station, TX, USA).

## Results

Of 623 participants included in the analyses, 359 (58 %) were HIV negative and 264 (42 %) were HIV positive. Participant characteristics are shown in Table 1. The median age was 51 years. The majority of the population was highly educated (55 %) and Dutch (73 %). The proportion of homosexual/bisexual men was significantly higher among HIV-positive than among HIV-negative participants (80 vs. 69 %, *P* = 0.004), and HIV-positive participants were more often married or cohabiting than HIV-negative participants (60 vs. 47 %, *P* = 0.001). Lifestyle was similar between HIV-positive and HIV-negative participants, except for smoking, with a median number of pack years of eight among HIV-positive and two among HIV-negative individuals (*P* = 0.002).

**Table 1:** Baseline characteristics of 623 working individuals aged 45–65 years by HIV status, participating in the AGE_h_IV Cohort Study (2010–2012)

	Total (*n* = 623)	HIV negative (*n* = 359)	HIV positive (*n* = 264)	*P* value
*Socio-demographics*				
Age (years) [median (IQR)]	50.6 (47.4–54.9)	50.6 (47.4–54.8)	50.7 (47.6–54.9)	0.96
Education [*n* (%)]				0.06
Low (until 16 years)	109 (17.5)	55 (15.3)	54 (20.5)	
Middle (until 18 years)	174 (27.9)	94 (26.2)	80 (30.3)	
High (≥college degree)	340 (54.6)	210 (58.5)	130 (49.2)	
Sexual preference				0.004
Homosexual/bisexual men	458 (73.5)	247 (68.8)	211 (79.9)	
Heterosexual men	89 (14.3)	57 (15.9)	32 (12.1)	
Women	76 (12.2)	55 (15.3)	21 (8.0)	
Ethnicity [*n* (%)]				0.47
Dutch	453 (72.7)	265 (73.8)	188 (71.2)	
Non-Dutch	170 (27.3)	94 (26.2)	76 (28.8)	
Marital status [*n* (%)]				0.001
Married/cohabiting	324 (52.0)	167 (46.5)	157 (59.5)	
Never married/divorced/widowed	299 (48.0)	192 (53.5)	107 (40.5)	
*Lifestyle*				
Physical activity [*n* (%)]				0.08
No	400 (64.2)	220 (61.3)	180 (68.2)	
Yes	223 (35.8)	139 (38.7)	84 (31.8)	
Smoking status [*n* (%)]				0.005
Never smoked	227 (36.4)	141 (39.3)	86 (32.6)	
Ever smoked	221 (35.5)	135 (37.6)	86 (32.6)	
Currently smoking	175 (28.1)	83 (23.1)	92 (34.9)	
Pack years of smoking [median (IQR)]	3.6 (0–21.0)	2.3 (0–18.0)	7.8 (0–27.5)	0.002
Heavy daily drinking [*n* (%)]				0.41
No	597 (95.8)	342 (95.3)	255 (96.6)	
Yes	26 (4.2)	17 (4.7)	9 (3.4)	
Use of cannabis/cocaine/ecstasy [*n* (%)]				0.82
No daily to monthly use	500 (80.3)	287 (79.9)	213 (80.7)	
Daily to monthly use	123 (19.7)	72 (20.1)	51 (19.3)	
*Work-related factors*				
Work type [*n* (%)]				0.02
Paid employment	455 (73.0)	256 (71.3)	199 (75.4)	
Entrepreneur	142 (22.8)	93 (25.9)	49 (18.6)	
Partly unfit for work	26 (4.2)	10 (2.8)	16 (6.1)	
Work hours/week [median (IQR)]	36 (30–40)	36 (30–40)	36 (32–40)	0.43
Lack of recovery opportunities, tertiles [*n* (%)]				0.17
Low	245 (39.3)	152 (42.3)	93 (35.2)	
Medium	213 (34.2)	114 (31.8)	99 (37.5)	
High	165 (26.5)	93 (25.9)	72 (27.3)	
Need for recovery, tertiles [*n* (%)]				0.25
Low	257 (41.3)	157 (43.7)	100 (37.9)	
Medium	187 (30.0)	107 (29.8)	80 (30.3)	
High	179 (28.7)	95 (26.5)	84 (31.8)	
*Absenteeism and comorbidities*				
Stayed home from work in past 6 months [*n* (%)]				0.91
Never	394 (63.2)	226 (63.0)	168 (63.6)	
1 time	156 (25.0)	92 (25.6)	64 (24.2)	
≥2 times	73 (11.7)	41 (11.4)	32 (12.1)	
Number of comorbidities [*n* (%)]				0.04
None	241 (38.7)	146 (40.7)	95 (36.0)	
1	240 (38.5)	143 (39.8)	97 (36.7)	
2	116 (18.6)	61 (17.0)	55 (20.8)	
≥3	26 (4.2)	9 (2.5)	17 (6.4)	
Moderate to severe depressive symptoms according to PHQ-9 scale [*n* (%)]				0.006
No	568 (91.2)	337 (93.9)	231 (87.5)	
Yes	55 (8.8)	22 (6.1)	33 (12.5)	
Score on the PHQ-9 scale [median (IQR)]	2 (1–5)	2 (1–4)	3 (1–6)	0.03
*HIV-related factors* ^a^				
Experienced HIV-related stigma at work [*n* (%)]				
No			237 (94.4)	
Yes			14 (5.6)	
Time since HIV diagnosis (years) [median (IQR)]			11.8 (6.0–16.3)	
Time since starting ART (years) [median (IQR)]			8.9 (3.9–13.9)	
CD4 count in year prior to enrollment (cells/mm^3^) [median (IQR)]			593 (450–787)	
Nadir CD4 count (cells/mm^3^) [median (IQR)]			190 (100–260)	
Prior clinical AIDS [*n* (%)]				
No			185 (73.7)	
Yes			66 (26.3)	
Undetectable viral load in year prior to enrollment [*n* (%)]				
No			11 (4.4)	
Yes			240 (95.6)	

*IQR* interquartile range, *ART* antiretroviral therapy

^a^Among a subset of 251 HIV-positive participants because of missing data

Overall, participants reported a median of 36 h of paid work per week. There were no differences in lack of recovery opportunities or need for recovery between HIV-positive and HIV-negative participants. Almost two-thirds reported never having stayed home from work in the past 6 months. HIV-positive participants were declared partly unfit for work twice as often as HIV-negative participants (6 vs. 3 %, *P* = 0.02), and they more often had 2 or ≥3 comorbidities (21 and 6 % in HIV-positive individuals vs. 17 and 3 % in HIV-negative individuals, respectively, *P* = 0.04). Lastly, HIV-positive participants more often had depressive symptoms (13 vs. 6 %, *P* = 0.006).

Among HIV-positive individuals, 6 % ever experienced HIV-related stigma at work. The median time since HIV diagnosis was 12 years. All participants were on cART, and the median time on antiretroviral therapy was 9 years. Median CD4 count was 593 cells/mm^3^, median nadir CD4 was 190 cells/mm^3^, and 26 % had prior AIDS. Almost all HIV-positive participants (96 %) had an undetectable viral load in the 12 months prior to enrollment.

### Determinants of work ability

Overall, 8 % of participants reported insufficient work ability. Although HIV-positive participants showed a higher proportion of insufficient work ability (9 %) than HIV-negative participants (7 %), this difference was not statistically significant in univariable analyses [odds ratio (OR) 1.46, 95 % confidence interval (CI) 0.81–2.62; Table 2]. The mean overall reported work ability was 7.7 (SD 1.6). Univariable analyses revealed higher proportions of insufficient work ability among participants with low education, women, and participants of non-Dutch ethnicity. A higher number of pack years of smoking was significantly associated with a higher prevalence of insufficient work ability. Participants who reported being partly unfit for work and who scored high on lack of recovery opportunities or need for recovery after work were more likely to have insufficient work ability. A higher number of weekly work hours was associated with a lower prevalence of insufficient work ability. Higher proportions of insufficient work ability were found among participants with depressive symptoms and among participants who stayed home from work ≥2 times in the past 6 months. Of the HIV-related factors, only HIV-related stigma at work was significantly associated with insufficient work ability (OR 4.34, 95 % 1.25–15.10 compared with participants who did not experience work-related stigma, Table 3).

**Table 2:** Univariable and multivariable analyses of determinants of work ability among 623 working individuals aged 45–65, participating in the AGE_h_IV Cohort Study (2010–2012)

	*n* insufficient work ability/*n* total (%)	OR (95 % CI)	*P* value	aOR (95 % CI)	*P* value	aOR (95 % CI)	*P* value
Total	49/623 (7.9)						
*Socio-demographics*				*Socio-demographics* ^a^			
HIV status			0.20		0.22		
Negative	24/359 (6.7)	1		1			
Positive	25/264 (9.5)	1.46 (0.81–2.62)		1.48 (0.80–2.73)			
Age (years, per 10-year increment)		1.41 (0.80–2.50)	0.24	1.37 (0.76–2.50)	0.30		
Education			0.005		0.05		
High (≥college degree)	17/340 (5.0)	1		1			
Middle (until 18 years)	16/174 (9.2)	1.92 (0.95–3.91)		1.72 (0.83–3.54)			
Low (until 16 years)	16/109 (14.7)	3.27 (1.59–6.72)		2.54 (1.19–5.44)			
Sexual preference			0.05		0.14		
Homosexual/bisexual men	31/458 (6.8)	1		1			
Heterosexual men	6/89 (6.7)	1.00 (0.40–2.46)		0.78 (0.30–2.01)			
Women	12/76 (15.8)	2.58 (1.26–5.29)		2.03 (0.93–4.43)			
Ethnicity			0.01		0.07		
Dutch	28/453 (6.2)	1		1			
Non-Dutch	21/170 (12.4)	2.14 (1.18–3.88)		1.80 (0.96–3.39)			
Marital status			0.46				
Married/cohabiting	23/324 (7.1)	1					
Never married/divorced/widowed	26/299 (8.7)	1.25 (0.69–2.24)					
*Lifestyle*						*Lifestyle* ^b^	
Physical activity			0.11				
No	29/300 (9.7)	1					
Yes	20/323 (6.2)	0.62 (0.34–1.11)					
Pack years of smoking (per 10-year increment)		1.16 (1.01–1.33)	0.05			1.15 (0.99–1.33)	0.07
Heavy drinking			0.50				
No	46/597 (7.7)	1					
Yes	3/26 (11.5)	1.56 (0.45–5.40)					
Use of cannabis/cocaine/ecstasy			0.40				
No daily to monthly use	37/500 (7.4)	1					
Daily to monthly use	12/123 (9.8)	1.35 (0.68–2.68)					
*Work-related factors*						*Work-related factors* ^c^	
Work type			<0.001				0.02
Paid employment	29/455 (6.4)	1				1	
Entrepreneur	9/142 (6.3)	0.99 (0.46–2.15)				2.09 (0.84–5.19)	
Partly unfit for work	11/26 (42.3)	10.77 (4.54–25.56)				4.44 (1.52–12.93)	
Work hours/week (per 10-h increment)		0.46 (0.34–0.61)	<0.001			0.58 (0.41–0.80)	0.001
Lack of recovery opportunities (tertiles)			0.004				0.06
Low	9/245 (3.7)	1				1	
Medium	16/213 (7.5)	2.13 (0.92–4.92)				1.69 (0.66–4.29)	
High	24/165 (14.6)	4.46 (2.02–9.87)				3.04 (1.16–7.93)	
Need for recovery (tertiles)			<0.001				<0.001
Low	7/257 (2.7)	1				1	
Medium	10/187 (5.4)	2.02 (0.75–5.40)				1.61 (0.56–4.60)	
High	32/179 (17.9)	7.77 (3.35–18.06)				5.70 (2.28–14.26)	
*Absenteeism and comorbidities*						*Absenteeism and comorbidities* ^d^	
Stayed home from work in past 6 months			<0.001				<0.001
Never	19/394 (4.8)	1				1	
1 time	13/156 (8.3)	1.79 (0.86–3.73)				1.45 (0.64–3.25)	
≥2 times	17/73 (14.3)	5.99 (2.94–12.21)				5.85 (2.58–13.27)	
Number of comorbidities			0.66				
None	15/241 (6.2)	1					
1	21/240 (8.8)	1.44 (0.73–2.88)					
2	11/116 (9.5)	1.58 (0.70–3.55)					
≥3	2/26 (7.7)	1.26 (0.27–5.82)					
Moderate to severe depressive symptoms according to PHQ-9 scale			<0.001				<0.001
No	28/568 (4.9)	1				1	
Yes	21/55 (38.2)	11.91 (6.14–23.13)				13.36 (6.20–28.75)	

*OR* odds ratio, *aOR* adjusted odds ratio, *CI* confidence interval

^a^Model 1: multivariable model with socio-demographic factors with univariable *P* value <0.1, HIV status, and age

^b^Model 2: multivariable model with lifestyle factors with univariable *P* value <0.1, corrected for socio-demographic variables, HIV status, and age

^c^Model 3: multivariable model with work-related factors with univariable *P* value <0.1, corrected for socio-demographic variables, HIV status, and age

^d^Model 4: multivariable model with absenteeism and comorbidities with univariable *P* value <0.1, corrected for socio-demographic variables, HIV status, and age

**Table 3:** Univariable and multivariable analyses of HIV-specific determinants of work ability among 251 working HIV-positive individuals aged 45–65, participating in the AGE_h_IV Cohort Study (2010–2012)

	*n* insufficient work ability/*n* total (%)	OR (95 % CI)	*P* value	aOR (95 % CI)^a^	*P* value
Total	24/251 (9.6)				
*HIV-related factors*					
Experienced HIV-related stigma at work			0.04		0.01
No	20/237 (8.4)	1		1	
Yes	4/14 (28.6)	4.34 (1.25–15.10)		6.13 (1.49–25.26)	
Time since HIV diagnosis (per 10-year increment)		1.47 (0.77–2.82)	0.24		
Time since starting ART (per 10-year increment)		1.57 (0.74–3.36)	0.24		
CD4 count in year prior to enrollment (per 100 cells/mm^3^)		1.06 (0.89–1.27)	0.52		
Nadir CD4 count (per 100 cells/mm^3^)		1.10 (0.83–1.48)	0.51		
Prior clinical AIDS			0.20		
No	15/185 (8.1)	1			
Yes	9/66 (13.6)	1.80 (0.74–4.31)			
Undetectable viral load in year prior to enrollment			0.37		
No	2/11 (18.2)	2.20 (0.45–10.84)			
Yes	22/240 (9.2)	1			

*OR* odds ratio, *aOR* adjusted odds ratio, *CI* confidence interval

^a^Multivariable model corrected for socio-demographic variables and age

In multivariable analyses (Table 2) of socio-demographic factors (model 1), lower educated participants were more likely to report insufficient work ability than participants with a high education (aOR 2.54, 95 % CI 1.19–5.44). Women and participants of non-Dutch ethnicity were more likely to have insufficient work ability (aOR 2.03, 95 % CI 0.93–4.43 and aOR 1.80, 95 % CI 0.96–3.39, respectively), but these differences were not statistically significant. When studying lifestyle factors in model 2, corrected for socio-demographics, there was a borderline statistically significant effect of smoking on work ability (aOR 1.15, 95 % CI 0.99–1.33 per 10-pack-year increment). In model 3, where work-related factors were corrected for socio-demographics, individuals who reported being partly unfit for work had higher odds of insufficient work ability (aOR 4.44, 95 % CI 1.52–12.93). A higher number of work hours was associated with a lower prevalence of insufficient work ability (aOR 0.58, 95 % CI 0.41–0.80 per 10-h increment). There was a borderline significant effect of recovery opportunities on work ability (*P* = 0.06), with participants who experience a high lack of recovery opportunities being more likely to report insufficient work ability (aOR 3.04, 95 % CI 1.16–7.93). Participants within the highest tertile of need for recovery after work more often had insufficient work ability (aOR 5.70, 95 % CI 2.28–14.26). In model 4, participants staying home from work ≥2 times in the past 6 months or reporting depressive symptoms were more likely to have insufficient work ability (aOR 5.85, 95 % CI 2.58–13.27 and aOR 13.36, 95 % CI 6.20–28.75, respectively). In multivariable analyses of HIV-related factors correcting for all socio-demographic variables, HIV-related stigma at work was strongly associated with work ability among HIV-infected participants (aOR 6.13, 95 % CI 1.49–25.26; Table 3). In sensitivity analyses with work ability categorized into three categories (<6, 6–8, >8), results were similar (data not shown).

## Discussion

The prevalence of insufficient work ability in this study was 8 %. There were no significant differences in the proportion of insufficient work ability between HIV-positive and HIV-negative individuals. Twice as many HIV-positive as HIV-negative participants were declared partly unfit for work, but percentages were low in both groups. A low educational level, working fewer hours, being partly unfit for work, experiencing a high need for recovery after work, staying home from work ≥2 times in the past 6 months, and reporting depressive symptoms were associated with insufficient work ability, independent of HIV status. Among HIV-positive individuals, having experienced work-related stigma was associated with insufficient work ability.

The prevalence of 8 % insufficient work ability found in this study is considered relatively high. This percentage is higher than in a previous Dutch study of work ability among hospital physicians, where 4 % of the population reported insufficient work ability (Ruitenburg et al. 2012). An explanation for the observed difference may be found in our study population being over 45 years of age, as older age has previously been found to be associated with lower work ability (Alavinia et al. 2009; van den Berg et al. 2009). Indeed, the mean work ability of 7.7 in our study is comparable to a previous study of work ability among workers aged 45 years and older in the Netherlands (Koolhaas et al. 2014).

In contrast to our hypothesis, the reported work ability of HIV-positive participants did not differ significantly from HIV-negative participants. As we measured work ability in a group of HIV-positive individuals still participating in work at the age of 45 and above, our study group may be a selection of healthier people than those that already dropped out of the working process at a younger age (e.g., healthy worker effect). This might especially be true for this older HIV-positive group, as it was more common to be declared 100 % unfit for work in the Netherlands in the past than it is today (Van Oorschot and Boos 2000). Indeed, analyses among participants from the AGE_h_IV Cohort Study showed an unemployment rate of 37 % in older HIV-positive individuals versus 21 % in older HIV-negative individuals. HIV-positive individuals were especially more often 100 % unfit for work, and unemployment was associated with health-related problems (Stolte et al. 2013). An additional explanation for the comparable work ability may be that a higher proportion of HIV-positive than HIV-negative participants was found to be partly unfit for work and that HIV-positive participants may have switched to jobs or work hours that are more compatible with their health. This may have enabled the HIV-positive participants to be in balance with requirements at work.

In contrast to previous studies (Alavinia et al. 2009; van den Berg et al. 2009), age was not significantly associated with work ability in this study. Our study only included workers between the ages of 45–65 years, which may have resulted in too little variation in age to show effects on work ability. Comorbidities frequently associated with HIV were shown to decrease the chances of HIV-infected individuals maintaining employment in an earlier study (Dray-Spira et al. 2012). Although HIV-infected participants in our study had more comorbidities than HIV-negative participants, comorbidity was not associated with work ability. A recent study showed that especially after the age of 60, HIV-positive individuals experienced more comorbidities than HIV-negative individuals (Schouten et al. 2014). With a median age of 50.6 years, our study population may be too young and healthy to show an effect of comorbidities on work ability. This could also explain why HIV-related factors did not seem to play a role in work ability in this study, while these factors have been shown to be associated with unemployment in earlier studies (Dray-Spira et al. 2006; Oliva 2010; Dray-Spira and Lert 2007; Ezzy et al. 1999).

In concordance with previous studies, we found lower education (Monteiro et al. 2006; Mazloumi et al. 2012; Golubic et al. 2009) and absenteeism (Gustafsson and Marklund 2011) to be associated with insufficient work ability. Experiencing a high lack of recovery opportunities at work was associated with work ability in univariable analyses as in other studies (van den Berg et al. 2009; Alavinia et al. 2009), but there was a borderline significant effect in the multivariable model. It is therefore unclear whether lack of recovery opportunities is an independent predictor of insufficient work ability. A high need for recovery after work has been shown to be predictive of sickness absence (de Croon et al. 2003), but may also be important in functioning of individuals at work, as it was associated with work ability in this study. Like in previous studies (Sun et al. 2013; Ruitenburg et al. 2012), there was a strong association between depressive symptoms and work ability in our study. Unfortunately, the cross-sectional design of this study makes it impossible to establish causality. However, results from the second data wave of the AGE_h_IV Cohort Study are expected soon, which would make it possible to study work ability longitudinally. Having experienced HIV-related stigma at work was associated with insufficient work ability among HIV-positive participants. Stigmatization has been shown to be associated with work participation (Stolte et al. 2013) and to prevent HIV-positive individuals from obtaining employment (Liu et al. 2012), but our results suggest that it may also impact their self-perceived work ability. Previous studies have shown that work type and job demands are associated with work ability (Koolhaas et al. 2014; McGonagle et al. 2015; Alavinia et al. 2009). Unfortunately, we did not measure these factors in the baseline questionnaire. In additional analyses, we examined income as a possible predictor of work ability, as this may be a proxy for work type, but income was not associated with insufficient work ability. Future studies should measure work type and job demands more precisely, to see whether there is an effect on work ability.

A major strength is that this study is the first to examine the work ability of HIV-positive individuals, with a comparable control group of HIV-uninfected individuals. This enabled us to study the effect of HIV positivity on work ability, with the results being less influenced by preexisting differences in socio-demographic characteristics between HIV-positive and HIV-negative people. A limitation is that the study sample consisted mostly of men who have sex with men (MSM) of Dutch origin, and results may therefore not be generalizable to women, heterosexual men, or migrants. Also, as the number of people with insufficient work ability in this study was quite low, we were not able to include all determinants into one multivariable model. These small numbers may have also resulted in limited power to identify determinants of work ability. Future research should include a larger group and combine these factors into one model, to study which independent determinants are most important.

The group of PLWH in the Netherlands is aging, and the statutory retirement age is increasing, which stresses the need to continue monitoring the impact of comorbidities and HIV on work ability. Studying factors involved in work ability will help preserve and improve social participation. Findings of this study also indicate the importance of earlier identification of work-related problems among HIV-infected individuals to prevent dropout at a younger age. Future studies should therefore focus on work ability among younger HIV-positive individuals and on changes in work ability over time during the course of an HIV infection. Although no differences in work ability between HIV-negative and HIV-positive older workers were found, results could still be used for optimizing work ability. Current guidelines on HIV and work in the Netherlands take most determinants identified in this study into account (Expertisegroep hiv en arbeid 2012), but fatigue after work may for example be assessed in a more standardized manner by using the need for recovery scale.

In conclusion, this study showed that HIV-positive people who still participate in the labor process between ages 45–65 seem to function as well at work as HIV-negative people of a similar age, although HIV-positive individuals were more often partly unfit for work. Studying work ability among younger HIV-positive individuals and changes in work ability over time may be useful to see how working careers develop and when interventions to preserve and improve work ability are needed most.
